# Supplementary material for: Discrete mechanical growth model for plant tissue
Source: PLoS One. 2019 Aug 12;14(8):e0221059. doi: 10.1371/journal.pone.0221059 (PMC6690522; doi:10.1371/journal.pone.0221059)
Supplement: S1 File — The model was implemented using the C++ programming language. The software uses the Intel threading building blocks (TBB) runtime library as a parallelization environment (available in open source from http://www.intel.com/software/products/tbb/), and for visualization the CASH library from Rob J. de Boer and Alex D. Staritsky (available in open source from http://theory.bio.uu.nl/rdb/software.html). (ZIP) [file pone.0221059.s001.zip › CODE/README/README.pdf]

Thank you very much for your interest in our software. To help you get started we provide here the most important information.

Operation System

The program is written to rely on POSIX standards. If you use windows 10, then you may still use our software easily by installing the “Windows Subsystem for Linux” [1].

Dependencies

The software uses the Intel threading building blocks (TBB) runtime library as a parallelization environment (available in open source from [2]), and for visualization the CASH library from Rob J. de Boer and Alex D. Staritsky (available in open source from [3]).

How to run the Program

Install the required dependencies.

Unpack the zipped file.

Change directory to the source code.

Run the make script: run the command “make”

Find binary file “wurzel” in the folder.

To run the program, type “./wurzel”.

Description of Structure

Please compare the section “Numerical methods” of the paper with the file main.cpp.

A loop over time (from t = start to T) is performed. Each time step a growth step happens (in growthfunction.h) which leads to a new set of resting distances of springs. Then the mechanical equations are solved to quasi equilibrium. Also, when springs exceed a certain length, then the remeshing algorithm (remeshing.h) adds new mass points, springs and hinges.

Description of Main Files

main.cpp
here the main() function is located.

parameter.h
here the parameters are set. It is the main control point of the program.

deleteoldoutputs.h
here the old outputs are removed

openandallocate.h
here the memory is allocated and files are opened.

setzero.h
here the allocated memory is filled with zeros.

crashwarn.h
here the program checks if your input is problematic. If your input contains an obvious error, then the program will crash and output an explanation. If your input raises some other issue that is worth a warning, then the program will output one.

initmesh.cpp
here the mesh of the model is set. Mass points get coordinates and masses, and their neighbors, distance to neighbors... are specified.

loadstate.h
here an old state of a simulation can be loaded (e.g. after adding turgor pressure).

Initialoutput.h
here the main computational, numerical and experimental settings are outputted at the start of the simulation.

establishturgorpressure.h
if parameter savestate == 1, then a simulation is run where step by step turgor pressure is added in here.

growthfunction.h
here a growth step is computed. It results in a new set of resting distances between mass points.

savestatebytime.h
If parameter savestatebytime == 1, then the current state of the simulation is saved, and the simulation is terminated here.

mechanicstep.cpp
here the mechanical model is solved until the sum of forces on every mass point is below the convergence threshold.

verlet.h
here a new set of coordinates for the mass points is computed in a verlet integration step.

lineartransformation.h
to cancel out rigid body translation and rotation a linear transformation is employed here.

convposition.h
here the coordinates of the mass points are translated to integers for the display function.

cheader.h
contains the extern-C filter, which manipulates C header files to be compatible with C++ and allow linking C++ and C objects without modification to any of the original C code

display2D.c

here the display is computed.

generaloutput.h
at the end of every growth time step a info re simulation time is outputted.

freeandclose.h
here the allocated memory is freed and files are closed.

finaloutput.h
here the final output is made, the duration of the simulation and the actual time is.

Setting up an Numerical Experiment: Example

The main control point of the program is the file “parameter.h”. There the main parameters can be adjusted to perform numerical experiments.

1. choose a mesh:
open file parameter.h: set parameters: for instance: res = 0 (easiest setting to use the model without cells); Nmpver = 33 (# mass points in vertical direction); Nmphor = 77 (# mass points in horizontal direction).

2. add turgor pressure:
set loadstate = 0; set savestate = 1; set turgorpressure = 0.20; save file; type in command line “make” to build a new executable binary. To run the program type “./wurzel”.
Now you should see a display pop up, and in the command line you should see how step by step turgor pressure is added. This simulation will take approximately one minute (depending on you machine).

3. run growth experiment:
open file parameter.h: loadstate = 1; set savestate = 0; save file; as above: type in command line “make” … execute program “./wurzel”.
Now you can see from the output that the previously saved state of the model is loaded; and that the growth function of the model is used now. An asymmetric growth rate causes an elongation and bending of the system, and residual strain fields emerge.

Contact

For inquiries, reporting a bug, or to comment on our software please email:

louis.daniel.weise@gmail.com

References

(1) https://docs.microsoft.com/en-us/windows/wsl/install-win10

(2) https://www.threadingbuildingblocks.org/

(3) http://theory.bio.uu.nl/rdb/software.html
