# Supplementary figures and images for: Discrete mechanical growth model for plant tissue
Source: PLoS One. 2019 Aug 12;14(8):e0221059. doi: 10.1371/journal.pone.0221059 (PMC6690522; doi:10.1371/journal.pone.0221059)

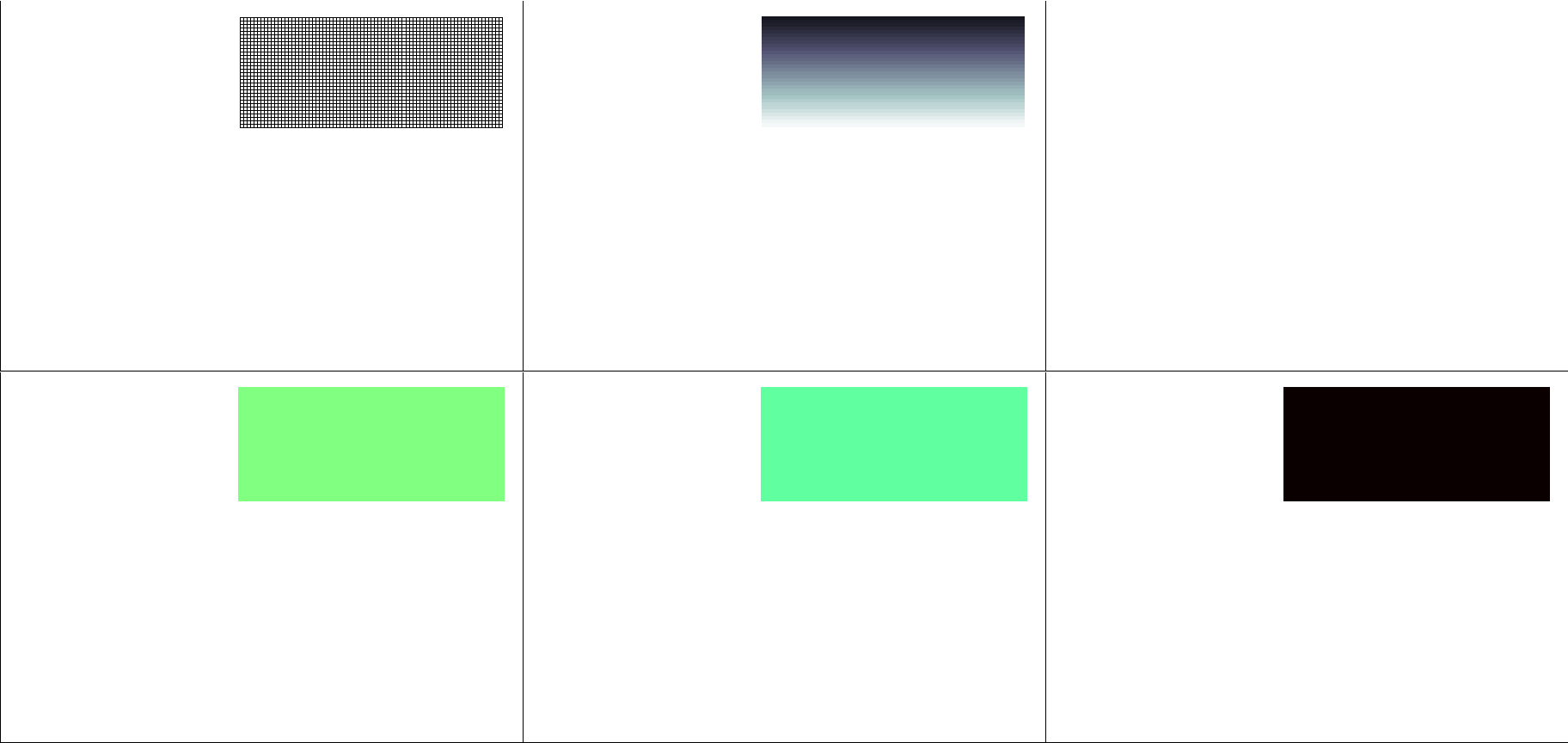

Supplement: S1 File — The model was implemented using the C++ programming language. The software uses the Intel threading building blocks (TBB) runtime library as a parallelization environment (available in open source from http://www.intel.com/software/products/tbb/), and for visualization the CASH library from Rob J. de Boer and Alex D. Staritsky (available in open source from http://theory.bio.uu.nl/rdb/software.html). (ZIP) [file pone.0221059.s001.zip › CODE/illustration1-root-bending/pictures/PNGs/temp/00000.png]

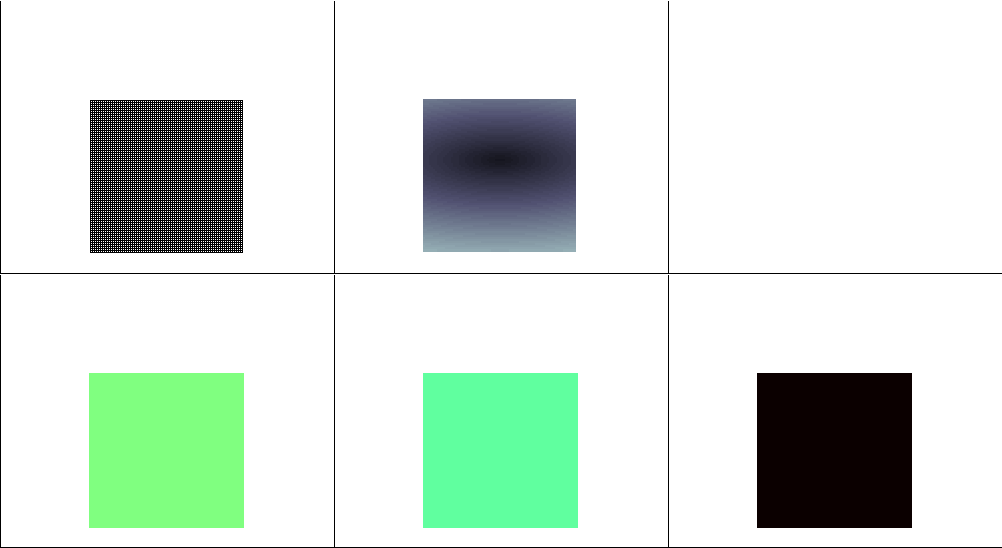

Supplement: S1 File — The model was implemented using the C++ programming language. The software uses the Intel threading building blocks (TBB) runtime library as a parallelization environment (available in open source from http://www.intel.com/software/products/tbb/), and for visualization the CASH library from Rob J. de Boer and Alex D. Staritsky (available in open source from http://theory.bio.uu.nl/rdb/software.html). (ZIP) [file pone.0221059.s001.zip › CODE/illustration2-leaf-growth/pictures/PNGs/temp/00000.png]
